# Comparison of Growth Performance, Immunity, Antioxidant Capacity, and Liver Transcriptome of Calves between Whole Milk and Plant Protein-Based Milk Replacer under the Same Energy and Protein Levels

**DOI:** 10.3390/antiox11020270

**Published:** 2022-01-29

**Authors:** Shuo Wang, Fengming Hu, Qiyu Diao, Shuang Li, Yan Tu, Yanliang Bi

**Affiliations:** 1 Key Laboratory of Feed Biotechnology of the Ministry of Agriculture and Rural Affairs, Institute of Feed Research, Chinese Academy of Agricultural Sciences, Beijing 100081, China; 82101185153@caas.cn (S.W.); hufengming@caas.cn (F.H.); diaoqiyu@caas.cn (Q.D.); 82101202361@caas.cn (S.L.); 2 Beijing Key Laboratory for Dairy Cow Nutrition, Institute of Feed Research, Chinese Academy of Agricultural Sciences, Beijing 100081, China

**Keywords:** calf, liver, milk replacer, plant protein, transcriptome

## Abstract

High-cost milk proteins necessitate cheaper, effective milk replacer alternatives, such as plant proteins. To examine plant protein-based milk replacer’s impact on growth performance, serum immune and antioxidant indicators, and liver transcriptome profiles in suckling calves. We assigned 28 newborn Holstein calves (41.60 ± 3.67 kg of body weight at birth) to milk (M) or milk replacer (MR) and starter diets pre-weaning (0–70 d of age) but with the same starter diet post-weaning (71–98 d of age). During the pre-weaning period, compared with the M group, MR group had significantly lower body weight, withers height, heart girth, average daily gain, feed efficiency, serum immunoglobulin (Ig) M concentration, superoxide dismutase concentration, and total antioxidant capacity; whereas they had significantly higher serum aspartate aminotransferase concentration. During the post-weaning period, MR group presented significantly higher average daily gain, alanine transaminase, aspartate aminotransferase, and malonaldehyde concentrations; whereas they had significantly lower serum IgA and IgM concentrations than the M group. Transcriptome analysis revealed 1, 120 and 293 differentially expressed genes (DEGs; MR vs. M group) in the calves from pre- and post-weaning periods, respectively. The DEGs related to xenobiotic and lipid metabolism and those related to energy metabolism, immune function, and mineral metabolism were up- and downregulated, respectively, during the pre-weaning period; during the post-weaning period, the DEGs related to osteoclast differentiation and metabolic pathways showed difference. In this study, compared with M group, MR group had the same growth performance during the overall experimental period; however, MR affected the hepatic metabolism, immune, and antioxidant function of calves. These observations can facilitate future studies on milk replacers.

## 1. Introduction

Successful calf production, especially dairy calf management, is crucial for the profitability and sustainability of the dairy industry [1,2]. Due to the underdeveloped rumen, suckling calves cannot meet their entire nutritional requirements through digesting solid feed; therefore, their nutrition primarily relies on milk and milk replacers [3]. Rearing calves using milk replacers has become a common practice in dairy farming worldwide. In the United States, more than 85% of calves are fed milk replacers before weaning [1]. The use of milk replacers, besides increasing the quantity of fresh milk available for human consumption, improves the health of the calves by avoiding disease transmission from the dam to the calf, reduces the cost of feeding, and provides farmers the opportunity to manipulate the nutrition of the calves for different production needs [4,5,6]. Therefore, an appropriate choice of milk replacer is essential for meeting the long-term targets of dairy farmers.

Protein is one of the main nutrients in the milk replacer, and the source as well as the concentration of the protein directly affects the growth and development of calves. In the 1980s, milk protein sources, including skim milk powder, casein, and whey protein, were the main protein sources in the milk replacers due to their balanced nutrition, fast digestion and absorption, and high utilization rate [7]. However, owing to the large-scale international trade of milk powder and the constant rise in the prices of casein and whey proteins, the use of plant proteins, especially the soy protein, in milk replacers is being studied extensively [8,9,10]. Compared with milk protein, plant proteins are high in crude proteins (CP) and functional amino acids [11]. However, compared to milk protein, the plant proteins have lower digestibility and solubility, poor amino acid profiles, and they contain anti-nutritional factors (ANF), such as protease inhibitors and phytic acid in soy protein, and non-starch polysaccharides in wheat protein [7,11,12]. Many studies have shown that the outcome of feeding calves with milk replacers containing plant protein in the pre-weaning period is generally unsatisfactory; however, it can increase the rate of rumen development and lower the weaning age through higher starter feed, increasing the average daily age (ADG) once the calves are weaned [5,8,13,14,15]. Meanwhile, wheat and rice proteins have been studied as potential replacements for milk protein in the formulation of milk replacers [16]. Wheat proteins, which contain glutamic acid and glutamine, are involved in the synthesis and metabolism of nucleic acids and proteins in intestinal cells; they also play a major role in intestinal mucosa cell regeneration and the maintenance of intestinal fitness [17]. Meanwhile, rice proteins, which contain lysine, methionine, and low ANF, possess antioxidative properties and regulates lipid metabolism [18,19]. To date, many studies have focused on the substitution of milk proteins with single plant proteins. Since the different kinds of plant proteins have different advantageous characteristics, we think that a combination of various plant proteins is necessary for the successful implementation of plant proteins in milk replacers.

As the metabolic powerhouse of the body, the liver is involved in the metabolism of the milk replacer and milk. Transcriptomics is a major tool in studying animal nutrition and health, and it is widely employed in biological system research [20,21]. Transcriptomics provides insights into the biological functions of genes that are upregulated or downregulated in experimental animals in response to the test materials [20,22]. Recently, with the development of RNA-Seq technology, transcriptomics has promoted genome research of biological systems, enabling the generation of biological information about experimental targets [22,23]. However, there have not been any studies on the gene expression in the livers of calves fed on milk replacers. Therefore, understanding the differentially expressed genes (DEGs) between the livers of milk-fed and milk replacer-fed calves might be helpful for future research on the effects of milk replacers on calves.

In this study, we hypothesized that the calves fed with a combination of various plant protein-based milk replacers have similar growth performance compared to whole milk-fed calves under the same energy and protein levels, which probably should be the case due to a better adaptability of their liver metabolism to milk replacer. Based on these hypotheses, this study aimed to examine the mechanism by which plant protein-based milk replacers influence the growth of the calves and identify possible hub-genes and molecular pathways involved in the metabolism of plant protein-based milk replacers using RNA-Seq technologies to characterize the liver transcriptome of the calves and provide the latest insights into the research and development of milk replacers.

## 2. Materials and Methods

### 2.1. Animal Ethics

This research was conducted in Chabei Pasture, Modern Farming, Zhangjiakou City, Hebei, China. The Animal Ethics Committee of the Chinese Academy of Agricultural Sciences (No. AEC-CAAS-20180224) approved the experimental procedures, and animal welfare and handling procedures were strictly followed during the experiment.

### 2.2. Animal, Study Design, and Diets

Twenty-eight newborn, female, and healthy Holstein calves (41.60 ± 3.67 kg of body weight at birth) were selected for this study. All calves were kept in individual calf pens (3 × 1.5 × 1.6-m^3^; length × width × height) and the bedding for calves was straw. The pens were cleaned and changed the straw before 1600 h every day to ensure the health and hygiene of the calves. Calves were drenched a total of 6 L of colostrum, with 4 L drenched within 1 h after birth and 2 L drenched 5 h after the first feeding. Before being drenched, colostrum quality was qualified (IgG > 55 g/L; total number of bacteria < 50,000 CFU/mL). During the pre-weaning period (2–70 d), the calves were fed liquid feed (whole milk or milk replacer) with 39 °C twice a day (08:00 and 18:00 h) using a bucket. Along with the liquid feed, the pelleted starter feed and clean water (39 °C) were provided ad libitum from 14 days of age. Subsequently, during the post-weaning period (71–98 d) the calves were fed only the pelleted starter feed.

These 28 calves were assigned to either the M or the MR group, according to their body weight at birth (mean body at birth of M vs. MR = 41.9 vs. 41.4 kg); each group consisted of 14 calves. Whole milk was obtained from milk tanks and pasteurized; the milk replacer was reconstituted as an emulsion (12.5%, *w*/*v*) using cooled (50–60 °C) boiled water (drinking water from pasture). After feeding with colostrum, the calves in the M group were fed on 8 L whole milk per day from 2 to 63 d, whereas the calves in the MR group were fed on 8 L whole milk per day from 2 to 7 d and then transitioned from whole milk to the plant protein-based milk replacer from 8 to 13 d (transition period). During the transition period, the ratio of milk replacer to whole milk was gradually increased from 1:2 to 2:1 (*v*:*v*). From 14 to 63 d, the calves in the MR group were fed on 8 L milk replacer per day. All the calves started weaning on 64 d, and the weaning period ended on 70 d. During the weaning period, the feeding amount of milk or milk replacer (according to their respective groups) was decreased 1 L per day. The milk replacer contained 50% plant proteins, including wheat, rice, and soybean protein. During the experimental period (lasting for 98 d), all the calves were reared in the same individual pens and fed the same starter feed. We had two rows of pens (12 individual pens per row) in this study, and all calves were randomly assigned to the pens. To calculate the daily matter intake (DMI) of each calf, the starter feed was changed at 08:00 h each day. The amounts of starter feed and milk or milk replacer that was remaining and the amount that was added were recorded. Moreover, the milk was sampled before the morning feeding every 15 d throughout the study period to measure its chemical composition according to the method described by Kong et al. [24]. The ingredients and the chemical compositions of the milk and milk replacer, and the starter feed are shown in Table 1 and Table S1, respectively.

### 2.3. Determination of Growth Performance and Serum Indicators

Body weights of the calves were recorded at birth and every 14 d thereafter before the morning feeding; additionally, other body measurements, including heart girth, withers height, and body length of the calves, were recorded before the morning feeding on 1, 70, and 98 d of age according to the method described by Kargar and Kanani [25]. ADG of the calves was calculated every 14 d. Feed efficiency was calculated as ADG/Total DMI (liquid DMI + starter feed DMI; kg/d). On 35, 42, 49, 84, 91, and 98 d of age, six healthy calves were selected from each group, and 10 mL of their blood samples was collected from the jugular vein before the morning feeding. Blood samples were centrifuged at 3000× *g* for 15 min (Tiangen OSE-MP25, Beijing, China) to obtain the serum, which was then stored in 1.5 mL centrifuge tubes at −20 °C for further analyses. The concentration of serum immunoglobulin (Ig) G, IgA, and IgM were determined using ELISA kits (F4042-A, F3995-A, and F6685-A, respectively). Aspartate aminotransferase (AST), Alanine transaminase (ALT), and alkaline phosphatase (ALP) concentrations were determined using an automatic biochemical analyzer (kehua-zy KHB-1280, Shanghai, China). The total antioxidant capacity (T-AOC), superoxide dismutase (SOD), glutathione peroxidase (GSH-PX), malondialdehyde (MDA), and catalase (CAT) concentrations in the serum were determined using commercial kits (Nanjing Jian Cheng Bioengineering Institute, Nanjing, China).

### 2.4. Liver Biopsy

Liver samples (the liver was sampled from the same calves from whom blood samples had been obtained) were collected from the calves (four calves per group) for liver biopsy using a handcrafted, stainless steel, biopsy gun according to the method described by Kong et al. [26]; samples were collected from the same eight calves (four calves per group) at 2 weeks before [liver samples from the M group before weaning (MBW) and liver samples from the MR group before weaning (RBW)] and after weaning [liver samples from the M group after weaning (MAW) and liver samples from the MR group after weaning (RAW)]. An approximately 15 cm^2^ skin area above each calf’s liver was shaved and disinfected with 75% ethyl alcohol. Thereafter, 10 mL of toluene thiazide hydrochloride (20 mg/mL) was injected subcutaneously and through the intercostal muscles at the sampling site. The sampling site was anesthetized, and a 1-cm incision was made using a sterile blade. Approximately 0.5 g of the liver tissue was collected into cryopreservation tubes at each sampling time using a handcrafted, stainless steel, biopsy gun for liver biopsy, and the liver tissue was stored at −80 °C for transcriptome assay.

### 2.5. Total RNA Extraction and Sequencing

The total RNA from the ground liver samples (total number of samples = 16) was extracted using TRIzol (Invitrogen, Carlsbad, CA, USA). The purity of RNA was determined using a Nano Drop^®^ND-1000 spectrophotometer (Implen, Santa Clara, CA, USA). The quality of the extracted RNA was assessed using an Agilent 2100 bioanalyzer (Agilent Technologies, Santa Clara, CA, USA). The RIN value of all samples was >8; 2 μg of RNA extracted from each sample was sent to Allwegene Technology Inc. (Beijing, China) for library building and sequencing. Briefly, mRNA was enriched using magnetic beads with Oligo(dT) and was broken into short fragments with fragmentation buffer. These fragments were reverse transcribed to cDNA for PCR amplification and cDNA library construction. After successful library construction, Illumina Hiseq 4000 PE150 (Illumina, San Diego, CA, USA) was used for sequencing.

### 2.6. Validation of RNA-Seq Data by Quantitative Real-Time PCR (qRT-PCR)

To verify the accuracy of RNA-Seq, we selected eight DEGs, including *CCND1*, *PCK1*, *ACACA*, *GSTM3*, *STAT3*, *MMP9*, *CYP4A22*, and *NAT10*. The IQ5 qRT-PCR detection system (Bio-Rad; Hercules, CA, USA) was used for the analyses of all the samples. The primers required for the *q*RT-PCR analyses of these eight DEGs were designed using NCBI primer-BLAST, and they were synthesized by Sangon Bioengineering (Shanghai, China) Co. Ltd. β-Actin was used as an internal control to normalize the expression data [27]. All the primers are listed in Table S2.

### 2.7. Statistical Analyses

Statistical analyses were conducted using GraphPad Prism 8 (GraphPad Software Inc., CA, USA) and R software (v 4.0.3). The statistical power for the serum samples of this study was >0.8 using the “Pwr” package (https://cran.r-project.org/web/packages/pwr/, accessed on 11 November 2021). Data were checked for normality using the shapiro.test function of the R software before analyses, and the data fitting non-normality were log transformed. Data on body weight, withers height, heart girth, and body length at 0 d of age were analyzed using *t*-test. Furthermore, at 70 and 98 d of age they were analyzed using AVCOVA as described by Wang et al [28]. The relevant linear model was used as follows:

$$Y_{ijk}=\mu +T_{i}+\alpha \left(R_{j}-\bar{R}\right)+e_{ijk}$$

where $Y_{ijk}$ is the dependent variable; μ is the average experimental value; $T_{i}$ is the fixed effect of treatments i (i = M or MR group); $\alpha \left(R_{j}-\bar{R}\right)$ designates the covariate variable of initial body weight, where the α is the regression coefficient relating initial body weight to the variable measured, $R_{j}$ is the initial body weight for the jth calf (j = 1, 2, 3, ……, 27, and 28), and $\bar{R}$ is the overall mean of the initial body weight; $e_{ijk}$ is the error term. If there was a co-effect in the progress of analyzing, we used the effects function in the ‘effects’ package (https://CRAN.R-project.org/web/packages/effects/, accessed on 11 November 2021) to remove covariate factors and to correct the average value of these indicators.

Data on intake, ADG, and feed efficiency were analyzed using mixed model in R. The relevant model used was as follows:

$$Y_{ijklm}=\mu +T_{ik}+D_{jk}+P_{k}+TD_{ijk}+TP_{ik}+Calf_{l}+e_{ijklm}$$

where $Y_{ijklm}$ is the dependent variable; μ is the average experimental value; $T_{ik}$ is the fixed effect of treatments i (i = M or MR group) during the period k (k = pre-weaning or post-weaning); $D_{jk}$ designates the repeated effect of the period k (k = pre-weaning or post-weaning); $P_{k}$ is the effect of period k (k = pre-weaning or post-weaning); $TD_{ijk}$ is the interaction effect of treatment and day during the period k (k = pre-weaning or post-weaning); $TP_{ik}$ is the interaction effect of treatment and period; $Calf_{l}$ is the random effect of lth calf (l = 1, 2, 3, ……, 27, and 28); $e_{ijklm}$ is the error term.

The data on serum indicators were analyzed using another mixed model in R. The relevant model used was as follows:

$$Y_{ijklm}=\mu +T_{ik}+D_{jk}+TD_{ijk}+Calf_{l}+e_{ijklm}$$

where $Y_{ijklm}$ is the dependent variable; μ is the average experimental value; $T_{ik}$ is the fixed effect of treatments i (i = M or MR group) during the period k (k = pre-weaning or post-weaning); $D_{jk}$ designates the repeated effect during the period k (k = pre-weaning or post-weaning); $TD_{ijk}$ is the interaction effect of treatment and day during the period k (k = pre-weaning or post-weaning); $Calf_{l}$ is the random effect of lth calf (l = 1, 2, 3, ……, 27, and 28); $e_{ijklm}$ is the error term.

The raw data of RNA-seq were processed with a Perl script. The clean data were obtained by removing reads containing adapters, reads containing poly-N, and reads of low quality from the raw data. The clean reads were then mapped to the bovine reference genome (http://bovinegenome.org, accessed on 2 November 2021) using the TopHat2 software (v 2.1.0). Gene expression levels in each library were normalized to fragments per kilobase of exon model per million mapped reads (FPKM). Differential gene expression analysis of the two groups (MR vs. M) was performed using the DESeq R package (1.10.1). Genes with *p*-value ≤ 0.05 and |log2(fold-change)| > 1.3 found by DESeq were assigned as DEGs (Kong et al., 2017; Papah et al., 2018). MCODE with standard parameters (node score cutoff, 0.2; K-Core, 2; maximum depth from seed, 100) from Cytoscape version 3.7.2 [29] was used for modular analysis. Gene Ontology (GO) and Kyoto Encyclopedia of Genes and Genomes (KEGG) for DEGs were analyzed using the CluoGo in Cytosacpe. STRING 11 (http://string-db.org/, accessed on 5 November 2021) was used to predict protein-protein interactions (PPI) of DEGs. A PPI network was drawn using Cytoscape and the cytoHubba application was used to identify the hub-genes [30,31].

The ‘corrplot’ package of R software was used to analyze the relationship between the DEGs and the apparent indicators that included feed efficiency and serum indicators (based on Spearman’s coefficient). All the data were reported as means, and the differences with a *p* < 0.05 were considered statistically significant.

## 3. Results

### 3.1. Intake and Growth Performance

The growth performance of the calves is shown in Table 2. There were no interactions among feeding group and day-age of the calves. The body weight, withers height, body length, and heart girth of the calves were not significantly different at birth (0 d) and 98 d of age. At 70 d of age, the calves in the MR group presented significantly lower body weight, withers height, and heart girth than those in the M group; however, body length was not significantly different between the two groups. During the pre-weaning period, the DMI of the liquid feed, ADG, and feed efficiency in the calves of the MR group were significantly lower compared to the calves of the M group; however, the DMI of the starter feed in the calves of the MR group were significantly higher than those in the M group. During the post-weaning period, the DMI of the starter feed and ADG in the MR group presented significantly higher levels than those in the M group; however, their feed efficiency was significantly lower relative to the M group. During the total experiment period (0–98 d), the calves of the MR group presented significantly higher overall DMI, yet showed a lower feed efficiency compared to the calves of the M group. However, the overall ADG was not significantly different between the two groups.

### 3.2. Serum Indicators

The serum indicators are shown in Table 3. Based upon our observations, there were no interactions between the feeding group (M or MR) and the day-age of the calves that would affect the concentrations of serum variables. The serum concentrations of ALP, IgG, GSH-PX, and CAT were not significantly affected in the calves of the MR group. However, during the pre-weaning period, the calves of the MR group showed significantly higher AST concentrations and significantly lower IgM, SOD, and T-AOC concentrations compared to the calves of the M group. Similarly in the post-weaning period, the calves of the MR group showed significantly higher ALT, AST, and MDA concentrations and lower IgA and IgM concentrations compared to the calves of the M group.

### 3.3. Mapping Summary Statistics

For each library, RNA-Seq produced more than 24,731,334 raw reads (Table S3). The sequencing generated 18,673,574–23,908,557 clean reads for each sample. The GC content in the libraries ranged from 51.49% to 52.41%. The 16 samples had at least 98.22% reads with ≥Q20, and 95.03% reads with ≥Q30. The majority of reads in each library were mapped to the bovine reference genome, and the average mapping rates were 92.58%, 93.59%, 93.83%, and 93.54% for the MAW, MBW, RAW, and RBW groups, respectively. Simultaneously, the MAW, MBW, RAW, and RBW groups had an average of 90.94%, 91.77%, 92.11%, and 91.72% reads mapped to the bovine genome, respectively.

### 3.4. Differentially Expressed Genes (DEGs)

**Figure 1:**
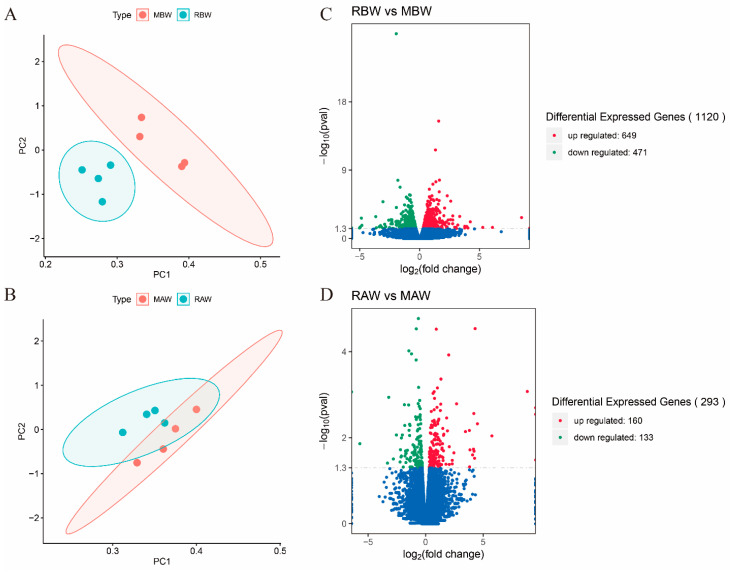
Gene expression between milk and milk replacer groups. (**A**) The principal component analysis (PCA) of all genes in liver between MBW and RBW. (**B**) The principal component analysis (PCA) of all genes in liver between MAW and RAW. (**C**) The expression of differentially expressed genes between two groups during pre-weaning period. (**D**) The expression of differentially expressed genes between two groups during post-weaning period. The red dots indicate upregulated differentially expressed genes and the green dots indicate downregulated differentially expressed genes. The blue dots reveal that there is no difference in the expression of genes between the two groups. MBW (liver samples from the milk group before weaning); RBW (liver samples from the milk replacer group before weaning); MAW (liver samples from the milk group after weaning); RAW (liver samples from the milk replacer after weaning).

The principal component analysis (PCA) of all genes from the liver samples showed separations between MBW and RBW or MAW and RAW based on the feeding group (M or MR) and the time of sample collection (pre-weaning or post-weaning) (Figure 1A,B). Compared to the gene expression in the MBW group, 1120 DEGs were found in the liver tissue of the RBW group, including 649 upregulated and 471 downregulated DEGs (Figure 1C). Similarly, 293 DEGs were identified in the liver samples of the calves in the RAW and MAW groups, among which 160 were upregulated and 133 were downregulated (Figure 1D). The heat plot was generated to visualize the distribution of the DEGs (Figure S1).

### 3.5. Enrichment Analysis of the DEGs

**Figure 2:**
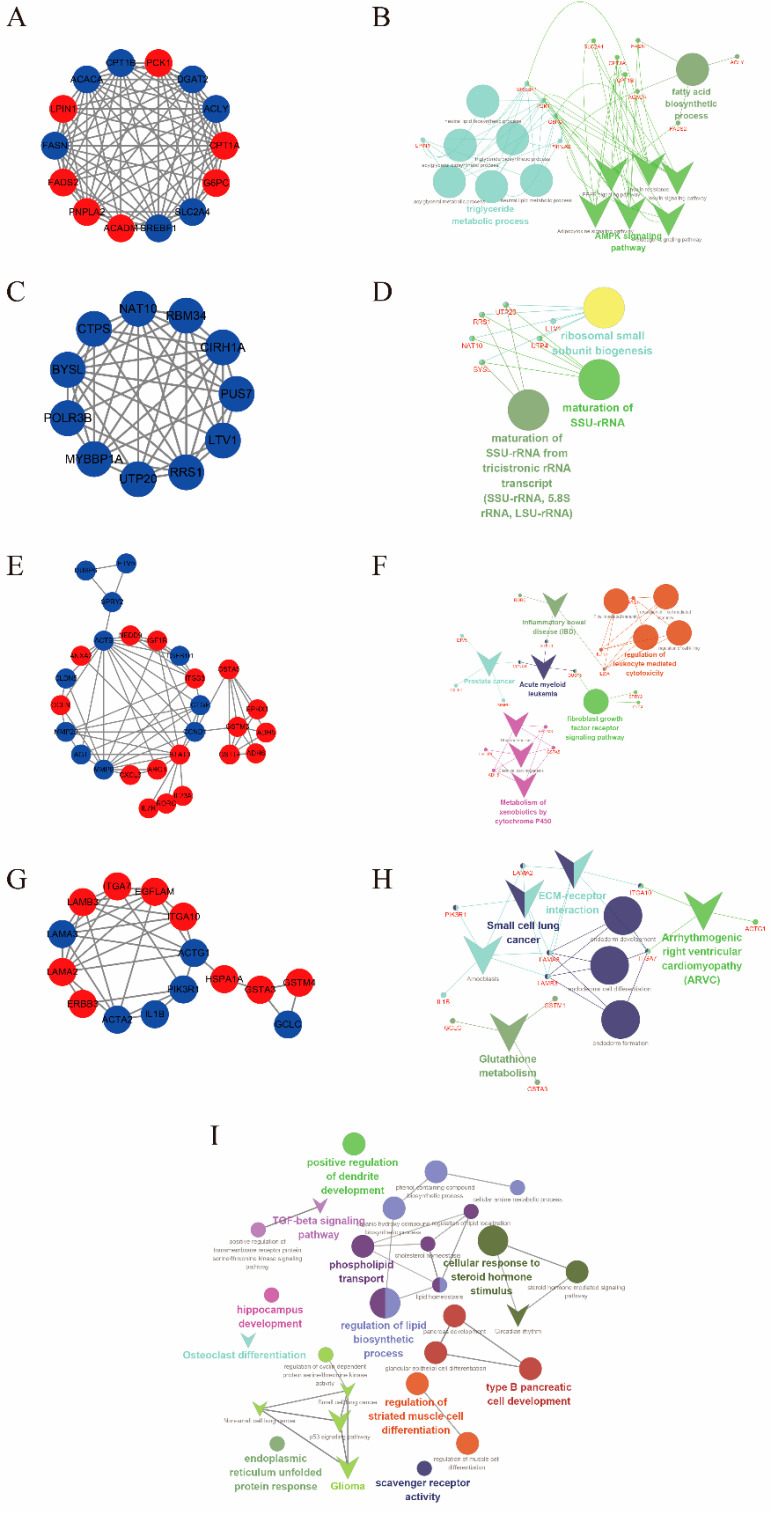
Enrichment analysis of differentially expressed genes (DEGs). (**A**,**B**) The gene interactive network and the enrichment result of Top 1 Module between RBW and MBW. (**C**,**D**) The gene interactive network and the enrichment result of Top 2 Module between RBW and MBW. (**E**,**F**) The gene interactive network and the enrichment result of Top 3 Module between RBW and MBW. (**G**,**H**) The gene interactive network and the enrichment result of Top 4 Module between RBW and MBW. (**I**) The enrichment result of all DEGs between RAW and MAW. Red represents the upregulated genes and blue represents the downregulated genes in the gene interactive network. Different colors represent different enrichment groups (based on the Cohen’s kappa score) in enrichment analysis. The triangle and circle graph represent the KEGG and GO results, respectively, in the enrichment analysis. MBW (liver samples from the milk group before weaning); RBW (liver samples from the milk replacer group before weaning). MAW (liver samples from the milk group after weaning); RAW (liver samples from the milk replacer group after weaning).

In the pre-weaning period, we found 25 clusters in 1120 DEGs using the MCODE between two groups (Supplementary File S2), of which the Top 4 clusters (based on network score, Figure 2A,C,E,G), including *ACACA*, *FASN*, *CPT1A*, *PCK1*, *SLC2A4*, *NAT10*, *ACTB*, *CCND1*, *GSTA5*, *ADH5*, and *ADH6*, among others, mainly enriched some functions including fatty acid metabolism, AMPK signaling pathway, xenobiotic metabolism, immunity and so on (Figure 2B,D,F,H). Then we analyzed the up- and down-regulated DEGs, respectively. Interestingly, the upregulated DEGs were primarily enriched in xenobiotic and lipid metabolism, including the metabolism of xenobiotics by cytochrome P450, chemical carcinogenesis, and the peroxisome proliferator-activated receptor (PPAR) signaling pathway, among others (Table S4). The downregulated DEGs were mainly enriched in energy metabolism, immune function, and mineral metabolism pathways, including mineral absorption, apoptosis, fatty acid biosynthesis, oxidoreductase activity, oxidation-reduction process, and NADH dehydrogenase activity, among others (Tables S4 and S5).

During the post-weaning period, osteoclast differentiation, muscle cell differentiation, and lipid metabolism appeared enrichment (Figure 2I). Similarly, we also analyzed the up- and down-regulated DEGs, respectively, and found that the metabolic pathway, Osteoclast differentiation, cell cycle, and cell projections appeared to have been enriched (Table S6; Figure S2).

### 3.6. Protein-Protein Interaction Analysis of DEGs Using STRING

**Figure 3:**
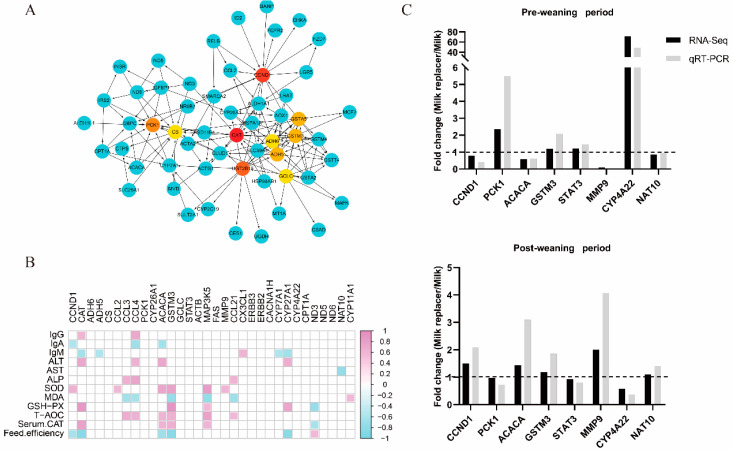
Relationship between hub-DEGs and phenotypes, and verification of transcriptome accuracy. (**A**) The top 10 hub-genes by cytoHubba in the top 200 differentially expressed genes between two groups. Blue genes are the first-stage genes of the hub-genes; (**B**) Correlation analysis between the apparent indicators and differently expressed genes. The pink color represents a positive correlation, and the blue color represents a negative correlation. The red to blue scale bar running from 1.0 to −1.0 represents r = 1.0 to −1.0. The blank represents *p* > 0.05. Serum immunoglobulin (Ig), glucose (GLU), triacylglycerol (TG), aspartate aminotransferase (AST), glutamic-pyruvic transaminase (ALT), alkaline phosphatase (ALP), total protein (TP), albumin (ALB), globulin (GLB), total antioxidant capacity (T-AOC), superoxide dismutase (SOD), glutathione peroxidase (GSH-PX), malondialdehyde (MDA), serum catalase (Serum CAT); (**C**) Validation of eight differentially expressed genes using quantitative real-time PCR (qRT-PCR). During pre- and post-weaning period, genes were validated. The mRNA level of each gene was normalized to that of β-actin (*n* = 4). The ratio of expression in livers of milk replacer calves to milk (Milk replacer/Milk) via RNA-Seq and RT-PCR is shown (*n* = 1).

We selected the top 200 DEGs (based on FC and *p*-value) to construct an interaction network (Figure S3). The genes in the center of the interaction network included *CCND1*, *CAT*, *ADH5*, *ADH6*, *CS*, *CCL2*, *PCK1*, *CYP26A1*, and *ACACA*. Additionally, we identified the top 10 hub genes (based on degree) among the top 200 DEGs, including *CAT*, *CCND1*, *UGT2B15*, *PCK1*, *ADH5*, *GSTA5*, *GSTM3*, *ADH6*, *GCLC*, and *CS*, using the cytoHubba application (Figure 3A).

### 3.7. Correlation Analysis between Hub-Genes among DEGs and Apparent Indicators

To identify the correlation between gene expression and the apparent indicators, we further analyzed Spearman’s rank correlation between the selected some key DEGs and the serum variables and the feed efficiency of the calves (Figure 3B). Interestingly, we observed that *CCND1*, *CAT*, *CCL3*, *CCL4*, *ACACA*, *GSTM3*, *MAP3K5*, *CYP27A1*, and *ND3* had correlations with the apparent indicators.

### 3.8. Validation of RNA-Seq Data by qRT-PCR

Furthermore, we validated the eight DEGs obtained from the pre- and post-weaning period samples using qRT-PCR technology. As seen in Figure 3C, the qRT-PCR results showed that the DEGs had the same expression pattern as observed in the RNA-Seq, indicating that our transcriptome data were highly reliable.

## 4. Discussion

Plant protein in calf milk replacer improves the quantity of animal protein available for human consumption, however plant protein-based milk replacer has a negative impact on calves. To our knowledge, this study is the first to systematically compare calves’ response between whole milk and plant protein-based milk replacer. The findings revealed that milk replacer had an influence on calves’ liver function, immunity, and antioxidant capacity, however had no effect on growth performance throughout the study.

The amount and type of the liquid feed are most likely to be the main factors that affect the solid feed intake of calves during the pre-weaning period. Previous studies have also shown that both restricted and unrestricted intake of milk as well as milk replacer can affect the solid feed intake of calves [15,32]. Zhang et al. found that the calves fed on milk replacers containing soy protein in the pre-weaning period had greater solid intake compared to the calves fed on whole milk [15]. Similarly, Ghorbani et al. reported that soymilk used as a novel milk replacer can stimulate early intake of starter feed in calves [5]. Likewise, in this study, lower liquid feed intake and higher solid feed intake were observed in the calves of the MR group compared to the calves of the M group in the pre-weaning period. Furthermore, we found lower ADG in the MR group, indicating that the calves may have better bioavailability of protein (casein, etc.) and energy (milk fat, lactose) from whole milk in comparison to the milk replacer. It is widely accepted that the plant proteins and the carbohydrates (glucose, etc.) in milk replacer cannot completely replace the milk proteins and lactose, respectively, of whole milk. In addition, there may be some unknown growth factors in whole milk that are responsible for the better ADG of the calves in the M group [33]. In this study, we discovered that the MR group had higher ADG after weaning than the R group. It is generally known that as calves digest solid feed, propionic acid and butyric acid are produced, which stimulate rumen development [34,35]. As a result, more solid feed consumption in the MR group will stimulate rumen development in advance before weaning, resulting in more energy from solid feed being available for growth after weaning. In addition, we also found that MR group could affect withers height and heart girth of calves. The calf stage is an essential period for bone growth and overall development, and calcium, phosphorus, and other minerals are also important for the development of bone [36]. In the present study, the mineral metabolism pathway was downregulated in the calves of the MR group; in particular, three genes (namely *ERBB2*, *ERBB3*, and *CACNA1H*) involved in calcium metabolism were downregulated in the calves fed on the milk replacer (Figure S4). Huang et al. reported that a plant protein-based milk replacer reduces calcium absorption [16]. In addition, Lee et al. reported that calves fed on milk replacers had a low rate of bone growth, similar with the observations of the present study [13]. Therefore, the outcome of this study indicates that exogenous mineral supplements, especially calcium, has a low bioavailability in the milk replacer; this suggests that we should pay attention to mineral element supplementation in future research related to the use of milk replacers for calves.

The liver is the largest metabolic organ of the body, and the health status of the liver is an indirect indicator of the health status of calves. Serum ALT and AST concentrations comprise a common indicator of hepatic health status. In the present study, during the pre-weaning period, the serum AST concentration in calves fed on the milk replacer was significantly higher than that of calves fed on whole milk. Therefore, we inferred that the milk replacer might have negatively affected the livers of the calves. Interestingly, TNF signaling and apoptosis pathway were significantly down-regulated, which also suggested MR might damage liver cell. Furthermore, we also found MR could influence immunity and antioxidant capacity in this study, which is similar to a previous study [16]. Seegraber et al. showed that replacing whey protein with plant proteins causes intestinal damage in suckling calves [37]. Moreover, the enrichment analysis of the liver DEGs during the pre-weaning period showed that the milk replacer regulated foreign material metabolism. Furtherly, we found that the key gene in foreign body metabolism, *GSTM3*, correlated with the serum antioxidant indicators. The GST genes are involved in the regulation of cellular glutathione. Moreover, studies have shown that FoxO signaling pathway was up-regulated, and the expression of *CAT* in the pathway involved in oxidative stress resistance and DNA repair [38], and it correlated with antioxidant indicators. As glutathione has been shown to be able to withstand oxidative stress in certain studies [39,40], the body boosts the expression of glutathione-related genes in response to oxidative stress damage. Milk replacer containing plant protein has been reported to contain ANFs, and these ANFs have adverse effects on the growth performance as well as the health of the calves [41,42]. Therefore, the observed reduction in the concentration of oxidation resistance indicators in the liver of the calves fed on the milk replacer can be attributed to the ANFs triggering oxidative stress. Similarly, the transcriptome analysis showed that *CCL2*, *CCL3*, *CCL21*, and *CX3CL1* were downregulated in the liver of the calves in the MR group. These DEGs are involved in the body’s immune response and have been previously detected in cattle [43]. Furthermore, these DEGs are involved in some pathways and BPs, including the TNF signaling pathway, chemokine activity, G protein-coupled receptor binding, and immune response, indicating that the milk replacer could reduce the immunity of the calves. Interestingly, we found that *CAT*, *CCL4*, and *CX3CL1* are also correlated with serum immunity indicators, suggesting that the decreased immunity of calves is related to oxidative stress. In short, based on the observations of the present study, we inferred that milk replacer might injure liver cells in calves, leading to oxidative stress and impaired immunity. However, there were no improvements in the concentrations of the serum indicators in the calves of the MR group during the post-weaning period, and the expressions of the related genes remained the same during the pre- and post-weaning periods, indicating that the liver function was not recovered.

Feed efficiency, which measures how efficiently animals use feed to produce meat, eggs, and milk, has traditionally been a key indication in livestock production. In this study, the calves of the MR group showed a lower feed efficiency compared to the calves of the M group, which is evident not only in milk replacer’s poor bioavailability and liver damage, but also in the expression pattern of genes associated to feed efficiency. The CYP genes play a significant role in feed efficiency, especially in functions related to liver metabolism [44,45,46]. Salleh et al. reported that *CYP11A1* upregulation results in declined efficiency of feed utilization in high residual feed intake dairy cattle. In the present study, *CYP27A1* has the same expression pattern as *CYP11A1* were upregulated and correlation with feed efficiency in the calves of the MR group [45]. In addition, the retinol metabolism pathway was significantly upregulated in the calves of the MR group compared to the calves of the M group. De Almeida Santana et al. had reported that retinol metabolism influences the feed conversion rate in cattle [47]. PPAR is an important fat metabolism pathway in the body. Interestingly, we found that bile acid, PPAR signaling, and fatty acid metabolism and degradation pathways were upregulated in the calves of the MR group; in contrast, the fatty acid biosynthesis pathway was downregulated, indicating that fat metabolism in calves is affected by feeding on milk replacers in the pre-weaning period. In the present study, we also observed that the upregulation of *STAT3* expression regulated the cell cycle and apoptosis via the JAK/STAT signaling pathway (Figure S5). *STAT3* stimulates fat deposition in cattle [48]. Therefore, the milk replacer used in the present study might have increased fat deposition in the liver, thereby negatively affecting the hepatic function. The protein encoded by *ACACA* plays an important role in converting acetyl CoA into fatty acids. Previous studies have shown that it is highly correlated with milk production, feed utilization, and fat deposition [49,50]. *FASN* is a rate-limiting gene in fatty acid synthesis, and it has a similar function to *ACACA*. *CPT1A* is important in regulating the oxidation of fatty acids in cells and transferring fatty acids from the outer membrane of the mitochondrion to the inner membrane [51]. Here, the GO cluster analysis showed that some BPs related to cellular respiration were downregulated. Oxidative phosphorylation occurs in the mitochondria and involves five complexes, which are essential for proton coupling and electron transfer [52,53]. In the present study, the expressions of *ND3*, *ND5*, *ND6*, and *NAT10* as well as the processes and pathways involved in cellular respiration were lower in the calves of the MR group as compared to the calves of the M group during the pre-weaning period. NADH dehydrogenase, which belongs to the NADH-CoQ dehydrogenase (complex I) family, is an enzyme that catalyzes the electron transfer from NADH to co-enzyme Q in the mitochondrial lining [54]. NADH is a marker in the mitochondrial chain producing energy. In the present study, downregulation of the gene regulating NADH dehydrogenase increased the NADH level in the mitochondria, thus affecting mitochondrial metabolism. The effects of Ca^2+^ on mitochondrial activities have been widely studied. Calcium plays an important role in regulating mitochondrial functions and ATP synthesis at different levels in organelles [55]. The Ca^2+^ concentration in the mitochondrial matrix has considerable effects on ROS generation, cytochrome C, and apoptosis [55]. Furthermore, *CS*, which plays an important role in regulating the citrate synthase activity in cells, was downregulated in the calves of the MR group. These results indicate that feeding on milk replacers can cause mitochondrial dysfunction in the calves. PPAR is closely related to the CYP genes. McCabe et al. reported that the expression of *CYP11A1* is upregulated during negative energy balance in cows, indicating that it plays an important role in the regulation of lipid and cholesterol synthesis in the liver [56]. Moreover, *CYP7A1* and *CYP27A1* are important regulators of cholesterol metabolism, bile acid biosynthesis, and steroid hormone pathways. Steroid hormone biosynthesis is an important metabolic pathway of negative energy balance in dairy cows, and this pathway was significantly enriched (RBW vs. MBW) in the present study. Pang et al. reported that energy-restricted feeding could lead to testis injury in sheep by affecting the process of cell apoptosis [57]. Therefore, a lack of energy might be harmful to the hepatic function in the calves of the MR group. We believe that the low energy of calves was linked to the content and source of fat present in the milk replacer. Although some studies have reported that higher fat content in milk replacers would influence the mammary gland development of heifers [58,59], Hu et al, have been reported there were no negative effect on growth performance of calves using palm oil and coconut oil instead of milk fat in milk replacer [60]. Therefore, the interaction between the source and concentration of fat for milk replacer may be the key factor affecting the performance of calves, which warrants further research.

Compensatory growth is a catch-up mechanism in animals, whereby an animal that had previously been affected by nutrient deficiency experiences accelerated growth when provided with feed that meets its nutritional requirements [61]. The greater ADG in the MR group in this study might be due to compensatory growth, which could be linked to the activation of the osteoclast growth-related pathway and gene expression of *CCND1*, *MMP9*, etc., in the post-weaning period. In addition, this compensatory growth may be attributed to the normalization of the mineral content and the energy metabolism in the calves in the post-weaning period. In domestic ruminants, rationally using compensated growth can improve the feed efficiency and meat quality of beef cattle and sheep [62,63,64]. However, as a result of the different concerns, many studies have found that compensated growth in human childhood could increase the risk of death and illness in adults [65,66,67]. Unfortunately, the long-term influence of compensatory growth in young ruminants is still unclear. It is well known that dairy cows have a long-life cycle, which is highly beneficial for humans; thus, we must pay adequate attention to the influence of compensatory growth on the milk yielding potential of dairy cows in the later stages of their lives. Within a reasonable range of ADG, lower ADG of suckling calves implies lower milk yield of these individuals later in their life [2]. According to the current research results, milk replacers containing plant protein can reduce the ADG of suckling calves, thus making it a controversial topic in the field of plant protein-based milk replacers. The shortage of protein resources is a global problem, especially in China, where high-quality animal proteins such as meat, eggs, and milk are in high demand and so, commercialized at a high price. Meanwhile, milk powder accounts for the largest share in world trade [9]. Therefore, in China, the consumption of milk (milk source protein) by suckling calves must be minimized to meet the high demands of milk for human consumption. At the same time, plant protein milk substitutes can increase starter feed intake and lower the weaning age of the calves, and this is incredibly beneficial for the rumen development of the calves and the effective use of roughage in the post-weaning period [5,8,13,14,15]. Nevertheless, it is still unclear whether plant protein-based milk replacer has any effect on the milk yielding potentials of these calves, later in life; this is definitely worthy of further experimental research. Therefore, it is necessary to develop milk replacers containing appropriate plant protein sources under the pretext of the shortage of high-quality protein resources in the world, especially in China.

## 5. Conclusions

In the present study, we comprehensively examined the effects of a plant protein-based milk replacer on the growth performance, immunity, antioxidant capacity, and liver transcriptome in calves. The findings revealed that milk replacer had a negative effect on calves’ liver function, immunity, and antioxidant capacity, however had no effect on growth performance throughout the study. The study provides insights into the metabolic mechanisms in the calves fed on a plant protein-based milk replacer, and it can serve as a basis for future studies on milk replacers.

## Figures and Tables

**Table 1:** Ingredient and chemical composition of milk and milk replace.

	Treatment	
Items	Milk Powder ^1^	Milk Replacer ^2^
Chemical composition (% of DM except for Dry matter; mean ± SD)		
Dry matter	96.76 ± 0.61	96.32
Gross energy MJ/kg	24.81 ± 0.41	24.62
Crude Protein	27.01 ± 0.36	26.93
Ether extract	30.67 ± 0.53	16.28
Ash	5.58 ± 0.05	5.53
Calcium	1.06 ± 0.03	1.02
Phosphorus	0.75 ± 0.03	0.77

^1^ Milk powder was obtained by drying the fresh whole milk, which was sampled every 15 days. ^2^ The ingredient compositions are 5% wheat protein powder, 4% rice protein powder, 18% whole soy powder, 6% concentrate whey protein, 22% whole milk power, 20% high protein whey powder, 12% fat powder, 0.2% vitamin complex, 4% Trace elements and mineral complexes, and 8.8% soluble carrier, and the lysine, Threonine, Methionine, essential amino acid, nonessential amino acid of milk replacer are 1.43%, 1.24%, 0.91%, 9.91%, and 15.59%, respectively (% of DM). Per kg milk replacer (DM basis) contains 15,000 IU vitamin A, 5000 IU vitamin D, 50 mg vitamin E, 6.5 mg vitamin B1, 6.5 mg vitamin B2, 6.5 mg vitamin B6, 0.07 mg vitamin B12, 20 mg vitamin B5, 13 mg vitamin B3, 0.1 mg vitamin H, 10 ppm Cu, 100 ppm Fe, 40 ppm Mn, 40 ppm Zn, 0.5 ppm I, 0.3 ppm Se, 0.1 ppm Co.

**Table 2:** Growth performance of calves fed milk or milk replacer. (*N* = 14 per group).

	Treatment (T) ^1^			*p*-Value				
Items ^4^	MR	M	SEM	T	Days (D) ^2^	T × D	P ^3^	T × P
Pre-weaning (0–70 d)								
DMI of starter feed, kg/d	0.23	0.16	0.02	0.009	<0.001	0.601		
DMI of liquid feed, kg/d	1.00	1.05	0.01	0.043	<0.001	0.622		
Total DMI ^4^, kg/d	1.23	1.22	0.02	0.513	<0.001	0.742		
ADG, kg/d	0.83	0.92	0.02	0.007	<0.001	0.461		
Feed efficiency ^5^	0.67	0.76	0.03	<0.001	<0.001	0.731		
Post-weaning (70–98 d)								
DMI of starter feed, kg/d	2.79	2.28	0.10	<0.001	<0.001	0.231		
ADG, kg/d	1.09	0.97	0.03	0.024	<0.001	0.814		
Feed efficiency	0.39	0.43	0.01	0.006	<0.001	0.731		
Overall (0–98 d)								
Total DMI, kg/d	1.68	1.51	0.03	0.002			<0.001	0.004
ADG, kg/d	0.91	0.94	0.02	0.334			<0.001	0.193
Feed efficiency	0.45	0.54	0.01	<0.001			<0.001	0.241
Body weight, kg								
Initial (d 0)	41.4	41.9	1.65	0.781				
Weaning (d 70)	98.2	107.4	2.83	0.004				
Final (d 98)	128.1	132.7	3.68	0.225				
Skeletal growth								
Withers height, cm								
Initial (d 0)	77.7	76.0	0.93	0.072				
Weaning (d 70)	94.6	97.2	1.13	0.026				
Final (d 98)	98.7	101.3	1.30	0.055				
Heart girth, cm								
Initial (d 0)	77.3	76.1	0.98	0.233				
Weaning (d 70)	107.1	109.9	1.10	0.018				
Final (d 98)	116.9	118.2	1.35	0.341				
Body length, cm								
Initial (d 0)	69.8	69.4	1.06	0.702				
Weaning (d 70)	97.3	99.4	1.04	0.131				
Final (d 98)	105.5	106.5	1.73	0.584				

^1^ MR = milk replacer; M = milk. ^2^ For all variables, days of age were used as 14-d period. ^3^ P = calf phase (pre-weaning vs. post-weaning period). ^4^ Total DMI = total daily matter intake (starter feed intake + liquid feed intake); ^5^ Feed efficiency = ADG/Total DMI; Standard error of the mean (SEM).

**Table 3:** Serum variables as influenced by feeding milk versus milk replacer to calves. (*N* = 6 per group).

	Treatment (T) ^1^			*p*-Value		
Items	MR	M	SEM	T	Days (D) ^2^	T × D
ALT, U/L						
pre-weaning (35–49 d)	7.5	7.1	0.99	0.524	0.979	0.621
post-weaning (84–98 d)	19.9	15.2	1.05	0.035	0.837	0.179
AST, U/L						
pre-weaning (35–49 d)	121.6	91.5	2.88	0.028	0.778	0.632
post-weaning (84–98 d)	183.2	148.7	4.23	0.036	0.081	0.712
ALP, U/L						
pre-weaning (35–49 d)	159.6	149.4	7.93	0.205	0.730	0.453
post-weaning (84–98 d)	188.1	186.1	11.49	0.693	0.334	0.818
IgG, g/L						
pre-weaning (35–49 d)	9.9	10.2	0.50	0.942	0.706	0.579
post-weaning (84–98 d)	12.7	12.3	0.44	0.994	0.206	0.765
IgA, g/L						
pre-weaning (35–49 d)	1.3	1.6	0.04	0.571	0.002	0.442
post-weaning (84–98 d)	1.1	1.2	0.02	0.028	0.004	0.668
IgM, g/L						
pre-weaning (35–49 d)	2.1	2.8	0.06	0.012	0.725	0.971
post-weaning (84–98 d)	2.1	2.7	0.06	0.001	0.819	0.715
SOD, U/mL						
pre-weaning (35–49 d)	85.3	99.1	3.06	0.045	0.679	0.391
post-weaning (84–98 d)	128.3	125.8	2.26	0.278	0.138	0.442
MDA, nmol/mL						
pre-weaning (35–49 d)	7.2	5.9	0.23	0.035	0.673	0.229
post-weaning (84–98 d)	4.7	4.2	0.11	0.015	0.714	0.814
GSH-PX, U/mL						
pre-weaning (35–49 d)	824	837	26.48	0.595	0.115	0.415
post-weaning (84–98 d)	1173	1202	30.46	0.698	<0.001	0.458
T-AOC, U/mL						
pre-weaning (35–49 d)	6.2	7.1	0.16	0.005	0.541	0.818
post-weaning (84–98 d)	9.8	10.3	0.42	0.103	0.328	0.643
CAT, U/mL						
pre-weaning (35–49 d)	8.6	8.5	0.33	0.596	0.333	0.992
post-weaning (84–98 d)	11.4	12.1	0.48	0.286	0.215	0.710

^1^ MR = milk replacer; M = milk. ^2^ For all variables, days of age were used as 8-d period. Standard error of the mean (SEM), Serum immunoglobulin (Ig), aspartate aminotransferase (AST), glutamic-pyruvic transaminase (ALT), alkaline phosphatase (ALP), total antioxidant capacity (T-AOC), superoxide dismutase (SOD), glutathione peroxidase (GSH-PX), malondialdehyde (MDA), catalase (CAT).

## Data Availability

The datasets used and analyzed during the current study are available from the corresponding author (Y.B.) on reasonable request.

## Supplementary Materials

The following supporting information can be downloaded at: https://www.mdpi.com/article/10.3390/antiox11020270/s1. Supplementary File S1: Table S1. The composition and nutritional value of starter (dry matter basis). Table S2. Gene primers used in the study for quantitative RT-PCR. Table S3. Summary of the average statistics of the sequence quality and alignment information between two groups. Table S4. KEGG enrichment analysis of differentially expressed genes between RBW and MBW. Table S5. GO analysis (top 20) of downregulated genes of the liver of calves between RBW and MBW group. Table S6. KEGG enrichment analysis of differentially expressed genes between RAW and MAW. Figure S1. Heat-map showing the gene expression data of the significantly differentially expressed genes. Figure S2. GO analysis DEGs of the liver of calves between RAW and MAW group. Figure S3. STRING analysis shows that differentially expressed genes are involved in TOP 200 and predicted protein-protein interactions between RBW and MBW. Figure S4. Sketch map of calcium signaling pathway between RBW and MBW group. Figure S5. Sketch map of JAK-STAT signaling pathway RBW and MBW group. Supplementary File S2: MCODE results between RBW and MBW. Supplementary File S3: The detail information of DEGs between RBW and MBW. Supplementary File S4: The detail information of DEGs between RAW and MAW.
