# Supplementary material for: Comparison of Growth Performance, Immunity, Antioxidant Capacity, and Liver Transcriptome of Calves between Whole Milk and Plant Protein-Based Milk Replacer under the Same Energy and Protein Levels
Source: Antioxidants (Basel). 2022 Jan 29;11(2):270. doi: 10.3390/antiox11020270 (PMC8868243; doi:10.3390/antiox11020270)
Supplement: Supplementary file 1 [file antioxidants-11-00270-s001.zip › Supplementary File S1.pdf]

Table S1. The composition and nutritional value of starter (dry matter basis).

Ingredients	Rate	Items	Nutritional value
Corn (%)	25	Dry matter, DM (%, air dry basis)	89.5
Extrude corn (%)	10	Crude protein, CP (%)	23.63
Wheat flour (%)	4.5	Crude fat (%)	4.98
Soybean meal (%)	25	Neutral detergent fiber (%)	16.56
Extruded soybean (%)	8	Acid detergent fiber (%)	6.78
Wheat bran (%)	6	Ash (%)	7.69
Soybean molasses (%)	7	Calcium (%)	1.51
Distillers dried grain and soluble (%)	5	Phosphate (%)	0.96
Glucose (%)	2	GE/(MJ/kg)	18.32
Dried whey powder (%)	3		
Limestone (%)	2.2		
$CaHPO_4$ (%)	0.5		
NaCl (%)	0.8		
Premix [1] (%)	1		

[1] The premix provided the following per kg of the starter: vitamin A 15000 IU, vitamin D 5000 IU, vitamin E 50 mg, Fe 90 mg, Cu 12.5 mg, Mn 60 mg, Zn 100 mg, Se 0.3 mg, Co 0.5 mg.

Table S2. Gene primers used in the study for quantitative RT-PCR.

Gene	Prime sequence 5'-3'	GenBank Accession no
PCK1	CCATCATTGACCCTG<br>AGAGCCTCGTAGACC	NM_174737.2
CCND1	GCCGAGGAGAACAAGCAG<br>GTCAGGCGGTGATAGGAG	NM_001046273.2
ACACA	TGGTGGGAATAAAGTG<br>TGGAAGAGTTGGGATA	NM_174224.2
GSTM3	GCTACAACCCTGACCACGAA<br>GGCACTTGGGCTCAAACATA	NM_001046560.1
STAT3	GCGATGGAGTATGTGC<br>CTTTCGTGTTTGTGCC	NM_001012671.2
MMP9	CGCTATGGCTACACTCCT<br>GTGGGCATCTCCCTGAAT	NM_174744.2
CYP4A22	TTCATCAATTCCCGTCTC<br>CTCCAATAGCAGCAAACC	NM_001098990.1
NAT10	CCGCTGAGAACAAGACCACG<br>AATCCAGGCACAGCAAGTCG	NM_001098031.2
β-actin	ATGATATTGCTGCGCTCGTG<br>CCGTGCTCAATGGGGTACTT	NM_173979.3

Table S3. Summary of the average statistics of the sequence quality and alignment information between two groups.

Sample	Total reads	Raw Reads	Clean Reads	Q20	Q30	GC Content	Uniquely mapped	Total mapped
MAW1	46648134	30591430	23324067	98.32%	95.33%	51.49%	42899582 (91.96%)	43612430 (93.49%)
MAW2	46612130	31156645	23306065	98.21%	95.13%	51.58%	41143372 (88.27%)	41884672 (89.86%)
MAW3	46616304	30964128	23308152	98.31%	95.29%	52.09%	42681774 (91.56%)	43486822 (93.29%)
MAW4	48920386	32866404	24460193	98.34%	95.35%	51.90%	45001964 (91.99%)	45828284 (93.68%)
MBW1	44846768	29602801	22423384	98.32%	95.31%	51.73%	41177208 (91.82%)	42052568 (93.77%)
MBW2	49010088	32834585	24505044	98.23%	95.03%	52.10%	44782448 (91.37%)	45621604 (93.09%)
MBW3	42137578	27946101	21068789	98.22%	95.03%	51.81%	38620962 (91.65%)	39395290 (93.49%)
MBW4	37358748	24731334	18679374	98.33%	95.32%	52.41%	34458854 (92.24%)	35124766 (94.02%)
RAW1	48934112	32254260	24467056	98.28%	95.23%	51.91%	44768604 (91.49%)	45546906 (93.08%)
RAW2	51361650	34557013	25680825	98.38%	95.43%	51.97%	47577256 (92.63%)	48421868 (94.28%)
RAW3	50138218	32722485	25069109	98.23%	95.10%	51.86%	46147780 (92.04%)	47087710 (93.92%)
RAW4	46662016	31272754	23331008	98.24%	95.04%	52.23%	43062342 (92.29%)	43883548 (94.05%)
RBW1	46680024	31284824	23340012	98.32%	95.32%	51.91%	42708316 (91.49%)	43666300 (93.54%)
RBW2	46692260	31150104	23346130	98.26%	95.18%	51.92%	42998928 (92.09%)	43783558 (93.77%)
RBW3	47817114	33243440	23908557	98.26%	95.19%	51.87%	43719936 (91.43%)	44617978 (93.31%)
RBW4	46249580	30567655	23124790	98.27%	95.19%	51.90%	42485202 (91.86%)	43277646 (93.57%)

Table S4. KEGG enrichment analysis of differentially expressed genes between RBW and MBW. (N=4 per group).

Pathway name	ID	*P*-value	Genes
The top 15 enrichment pathway based on the upregulated DEGs (RBW vs MBW)			
Metabolism of xenobiotics by cytochrome P450	bta00980	8.7E-09	LOC540544 ADH5 SULT2A1 Novel02331 ADH6 LOC100138004 HSD11B1 GSTA5 GSTA3 Novel01381 GSTM1 GSTA2 EPHX1 ENSBTAG00000039362 AKR7A2 GSTM3
Chemical carcinogenesis	bta05204	5.3E-08	LOC540544 ADH5 SULT2A1 Novel02331 ADH6 GSTA2 HSD11B1 GSTA5 CYP3A5 GSTA3 ENSBTAG00000015775 GSTM1 EPHX1 ENSBTAG00000039362 LOC100138004 GSTM3
Drug metabolism - cytochrome P450	bta00982	1.8E-07	FMO5 LOC540544 ADH5 GSTM3 Novel02331 GSTA3 GSTA2 AOX1 ADH6 GSTA5 FMO1 GSTM1 LOC100138004 ENSBTAG00000039362
Steroid hormone biosynthesis	bta00140	4.6E-06	LOC540544 AKR1D1 Novel02331 STS HSD11B1 CYP7A1 CYP3A5 COMT HSD17B2 HSD17B12 LOC100138004 ENSBTAG00000039362
Retinol metabolism	bta00830	2.4E-05	LOC540544 ADH5 ALDH1A1 ADH6 Novel02331 AOX1 CYP4A22 CYP3A5 RDH16 LOC100138004 ENSBTAG00000039362
Primary bile acid biosynthesis	bta00120	1.0E-04	HSD17B4 CYP27A1 SCP2 AKR1D1 CYP7A1 CYP39A1
Pentose and glucuronate interconversions	bta00040	2.4E-04	LOC540544 UGDH Novel02331 SORD LOC100138004 ALDH3A2 ENSBTAG00000039362
Ascorbate and aldarate metabolism	bta00053	4.1E-04	LOC540544 UGDH Novel02331 LOC100138004 ALDH3A2 ENSBTAG00000039362
PPAR signaling pathway	bta03320	6.4E-04	CPT1A CYP27A1 SCP2 CYP4A22 SCD CYP7A1 ENSBTAG00000047957 FADS2 ACADM PCK1
Starch and sucrose metabolism	bta00500	2.5E-03	LOC540544 UGDH Novel02331 G6PC TREH LOC100138004 ENSBTAG00000039362
Fatty acid metabolism	bta01212	3.1E-03	CPT1A HACD3 SCD ENSBTAG00000047957 FADS2 HSD17B12 ACADM
Biosynthesis of unsaturated fatty acids	bta01040	3.4E-03	FADS2 HSD17B12 ENSBTAG00000047957 SCD HACD3
FoxO signaling pathway	bta04068	4.9E-03	IRS2 FBXO32 INSR IGF1R GADD45A STAT3 CCNB3 CAT G6PC GADD45B IL7R PCK1
Fatty acid degradation	bta00071	6.0E-03	ADH6 CPT1A ADH5 CYP4A22 ACADM ALDH3A2
Platinum drug resistance	bta01524	1.1E-02	MSH2 GSTM3 ERBB2 REV3L GSTA2 GSTA5 GSTA3 GSTM1

The top 15 enrichment pathway based on the downregulated DEGs (RBW vs MBW)			
Mineral absorption	bta04978	3.2E-03	SLC40A1 HMOX1 MT2A SLC30A1 HMOX2 ATP1B2
Pyrimidine metabolism	bta00240	5.6E-03	CANT1 Novel01289 POLR3E Novel00553 NME2 POLR3B CTPS1 POLR3H UPP1
Apoptosis	bta04210	5.9E-03	CTSC TUBA1C TNFRSF10D FAS CTSZ ENSBTAG00000047389 PIK3R1 ACTG1 ACTB MAP3K5 TUBA8
Influenza A	bta05164	1.0E-02	PYCARD TNFRSF10D IL1B CCL2 ENSBTAG00000047389 GSK3B PIK3R1 ACTG1 ACTB TMPRSS4 FAS MAP2K3
Fatty acid biosynthesis	bta00061	1.1E-02	ACACA ACSL3 FASN
Maturity onset diabetes of the young	bta04950	1.1E-02	FOXA3 ONECUT1 HNF1A HES1
TNF signaling pathway	bta04668	2.4E-02	IL1B CX3CL1 MMP9 CCL2 PIK3R1 MAP3K5 FAS MAP2K3
Steroid biosynthesis	bta00100	3.0E-02	FDFT1 CYP51A1 SQLE
Terpenoid backbone biosynthesis	bta00900	3.3E-02	HMGCR MVD HMGCS1
Signaling pathways regulating pluripotency of stem cells	bta04550	3.4E-02	ONECUT1 INHBE GSK3B INHBA FGFR2 HNF1A DVL2 FZD7 PIK3R1
Insulin signaling pathway	bta04910	3.4E-02	SREBF1 SORBS1 PPP1R3B SLC2A4 FASN GSK3B PIK3R1 ACACA PPP1R3C
Vitamin digestion and absorption	bta04977	3.7E-02	SLC19A2 LRAT SLC5A6
AMPK signaling pathway	bta04152	4.4E-02	SREBF1 HMGCR SLC2A4 FASN CCND1 PIK3R1 ACACA CPT1B
Cytosolic DNA-sensing pathway	bta04623	4.6E-02	POLR3H IL1B POLR3E POLR3B PYCARD
Salmonella infection	bta05132	5.2E-02	ACTG1 IL1B CCL3 PYCARD RILP ACTB

MBW (liver samples from the milk group before weaned); RBW (liver samples from the milk replacer group before weaned).

Table S5. GO analysis (top 20) of downregulated genes of the liver of calves between RBW and MBW group. (N=4 per group).

GO accession	Description	Term type	*P*-value	Gene
GO:0055114	oxidation-reduction process	Biological process	4.6E-04	HMOX1 CS KIAA1217 NAT10 HMOX2 ND6 ND5 COMMD3 ACLY
GO:0046912	transferase activity transferring acyl groups acyl groups converted into alkyl on transfer	Molecular function	5.3E-04	HMGCS1 ACLYCS
GO:0005125	cytokine activity	Molecular function	1.0E-03	CCL2 EIF2B2 CCL3 CX3CL1 CCL21
GO:0015980	energy derivation by oxidation of organic compounds	Biological process	1.1E-03	KIAA1217 NAT10 ND6 ND5 COMMD3 ACLY CS
GO:0045333	cellular respiration	Biological process	1.1E-03	CS ACLY COMMD3 ND5 ND6 NAT10 KIAA1217
GO:0003954	NADH dehydrogenase activity	Molecular function	2.6E-03	ND6 ND5 NAT10 KIAA1217
GO:0006120	mitochondrial electron transport NADH to ubiquinone	Biological process	2.6E-03	KIAA1217 NAT10 ND5 ND6
GO:0008137	NADH dehydrogenase (ubiquinone) activity	Molecular function	2.6E-03	KIAA1217 NAT10 ND5 ND6
GO:0050136	NADH dehydrogenase (quinone) activity	Molecular function	2.6E-03	ND6 ND5 NAT10 KIAA1217
GO:0001664	G protein-coupled receptor binding	Molecular function	2.9E-03	CX3CL1 CCL21 CCL2 CCL3
GO:0008009	chemokine activity	Molecular function	2.9E-03	CCL3 CCL2 CCL21 CX3CL1
GO:0042379	chemokine receptor binding	Molecular function	2.9E-03	CCL21 CX3CL1 CCL3 CCL2
GO:0016746	transferase activity transferring acyl groups	Molecular function	3.0E-03	SAT2PLA2G15NAT10HMGCS1ACLYCS
GO:0016655	oxidoreductase activity acting on NAD(P)H quinone or similar compound as acceptor	Molecular function	3.3E-03	KIAA1217 NAT10 ND6 ND5
GO:0004392	heme oxygenase (decyclizing) activity	Molecular function	3.5E-03	HMOX1 HMOX2
GO:0006787	porphyrin-containing compound catabolic process	Biological process	3.5E-03	HMOX1 HMOX2
GO:0006788	heme oxidation	Biological process	3.5E-03	HMOX1 HMOX2
GO:0033015	tetrapyrrole catabolic process	Biological process	3.5E-03	HMOX1 HMOX2
GO:0051187	cofactor catabolic process	Biological process	3.5E-03	HMOX1 HMOX2
GO:0004108	citrate (Si)-synthase activity	Molecular function	3.7E-03	ACLY CS

MBW (liver samples from the milk group before weaned); RBW (liver samples from the milk replacer group before weane.

Table S6. KEGG enrichment analysis of differentially expressed genes between RAW and MAW. (N=4 per group).

Pathway name	ID	*P*-value	Genes
The top 15 enrichment pathway based on the downregulated DEGs (RAW vs. MAW)			
Metabolic pathways	bta01100	3.4E-04	Novel01818 TPH1 LOC104968656 LDHA ND5 FUT4 UROS PLCE1 MRI1 ANPEP CYTB SYNJ2 NMNAT2 GBA3 ASPA
Bile secretion	bta04976	3.9E-03	SLC22A7 ABCG8 AQP1
TGF-beta signaling pathway	bta04350	6.1E-03	INHBC BMP5 INHBE
Circadian rhythm	bta04710	9.4E-03	CRY1 ARNTL
Thyroid hormone signaling pathway	bta04919	1.4E-02	PLCE1 ENSBTAG00000010814 THRB
Cysteine and methionine metabolism	bta00270	1.6E-02	MRI1 LDHA
Tryptophan metabolism	bta00380	1.8E-02	TPH1 LOC104968656
Hedgehog signaling pathway	bta04340	1.8E-02	DHH HHIP
Notch signaling pathway	bta04330	1.9E-02	Novel00962 MAML3
Parkinson's disease	bta05012	2.7E-02	ND5 CYTB GPR37
Inositol phosphate metabolism	bta00562	4.1E-02	PLCE1 SYNJ2
Phosphatidylinositol signaling system	bta04070	7.0E-02	PLCE1 SYNJ2
Selenocompound metabolism	bta00450	7.9E-02	LOC104968656
Proximal tubule bicarbonate reclamation	bta04964	9.4E-02	AQP1
Histidine metabolism	bta00340	1.1E-01	ASPA
The top 15 enrichment pathway based on the upregulated DEGs (RAW vs. MAW)			
Osteoclast differentiation	bta04380	3.0E-05	ENSBTAG00000019348 ENSBTAG00000039892 Novel03047 ENSBTAG00000000930 Novel03048 Novel01105 ENSBTAG00000039086
Antigen processing and presentation	bta04612	1.3E-03	CTSV ENSBTAG00000008959 Novel01758 IFI30
Viral carcinogenesis	bta05203	3.5E-03	CDKN2B Novel01758 CCND1 CDK6 ENSBTAG00000008959 ENSBTAG00000046865
Phagosome	bta04145	3.6E-03	TUBB2B CTSV ENSBTAG00000008959 TUBB2A Novel01758
Cell cycle	bta04110	8.7E-03	GADD45B CDKN2B CCND1 CDK6

Viral myocarditis	bta05416	9.2E-03	ENSBTAG00000008959 Novel01758 CCND1
p53 signaling pathway	bta04115	9.9E-03	GADD45B CCND1 CDK6
Oxytocin signaling pathway	bta04921	1.7E-02	RYR1 MYLK4 Novel01649 CCND1
Small cell lung cancer	bta05222	1.8E-02	CDK6 CDKN2B CCND1
Circadian rhythm	bta04710	1.8E-02	Novel00918 NR1D1
Graft-versus-host disease	bta05332	2.6E-02	ENSBTAG00000008959 Novel01758
Allograft rejection	bta05330	3.5E-02	ENSBTAG00000008959 Novel01758
Type I diabetes mellitus	bta04940	4.1E-02	ENSBTAG00000008959 Novel01758
Lysosome	bta04142	4.3E-02	DNASE2B CTSV GM2A
Autoimmune thyroid disease	bta05320	5.1E-02	ENSBTAG00000008959 Novel01758

MAW (liver samples from the milk group after weaned); RAW (liver samples from the milk replacer group after weane.

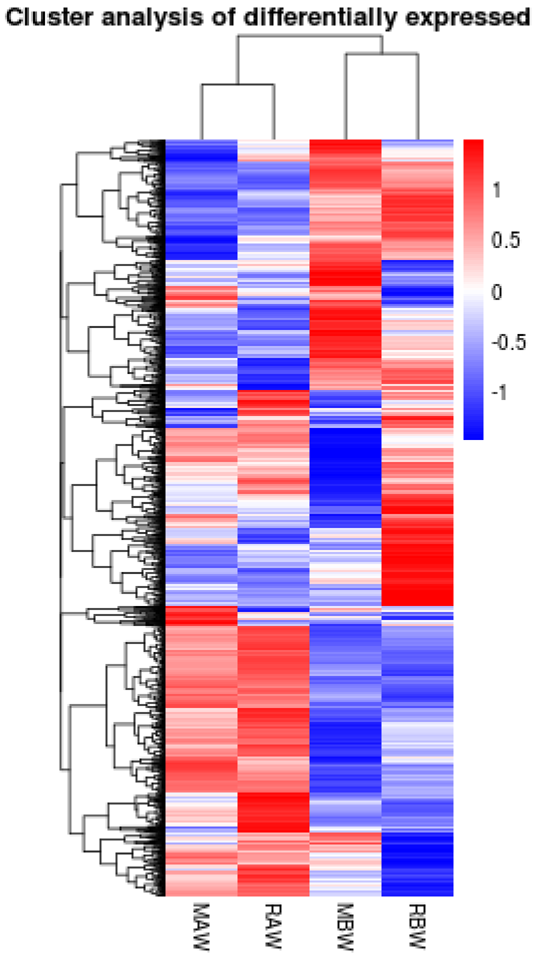

Figure S1. Heatmap showing the gene expression data of the significantly differentially expressed genes.

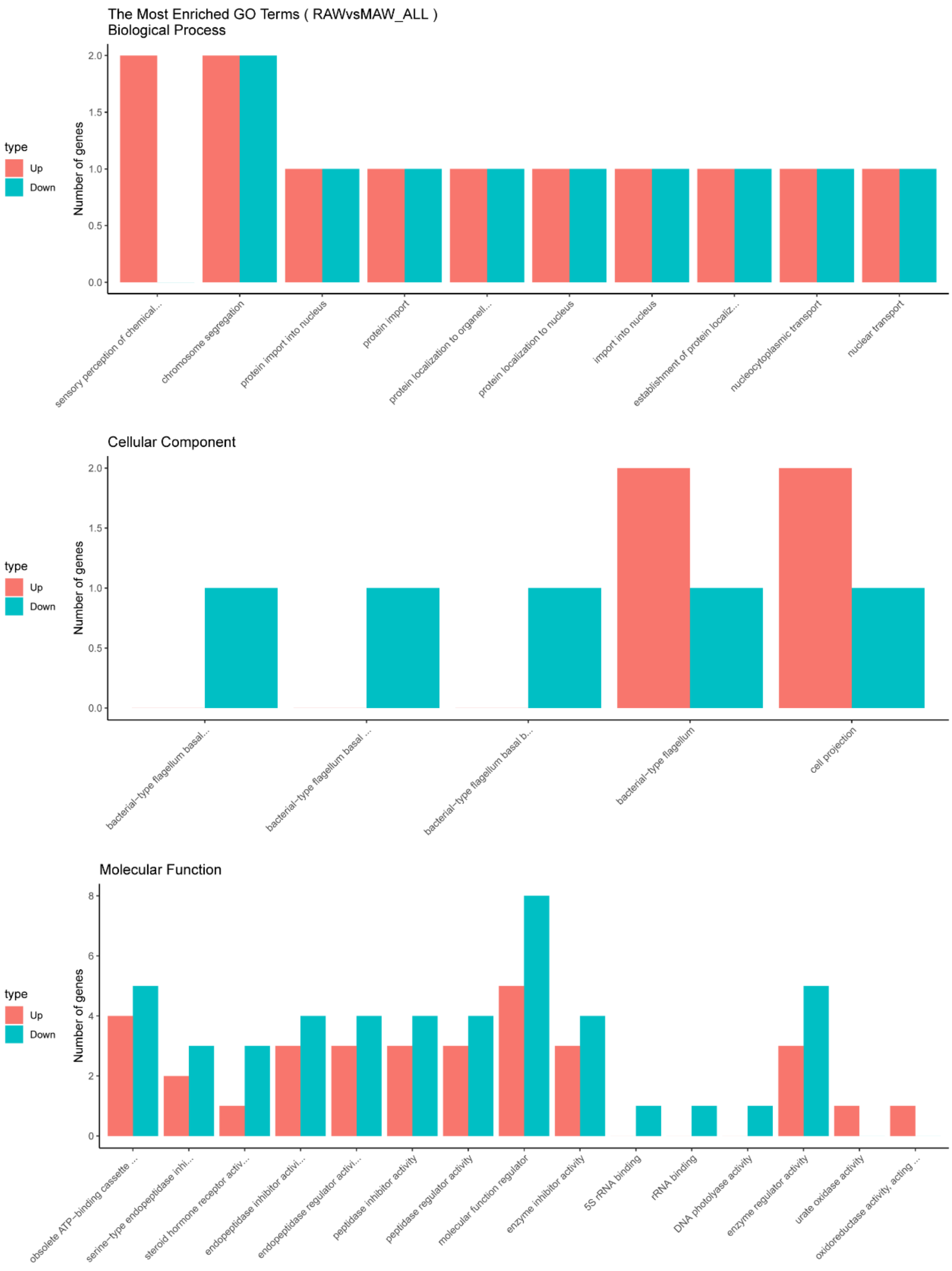

Figure S2. GO analysis DEGs of the liver of calves between RAW and MAW group.

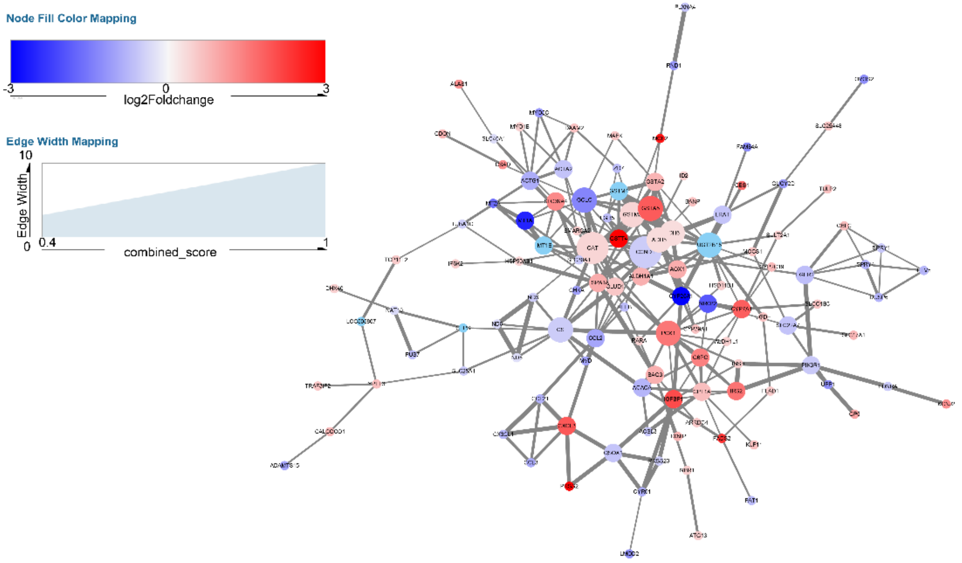

Figure S3. STRING analysis shows that differentially expressed genes are involved in TOP 200 and predicted protein-protein interactions between RBW and MBW. Circles of different sizes represent the significance of predicting associations. Red represents upregulated protein and blue represents downregulated protein. MBW (liver samples from the milk group before weaned); RBW (liver samples from the milk replacer group before weaned).

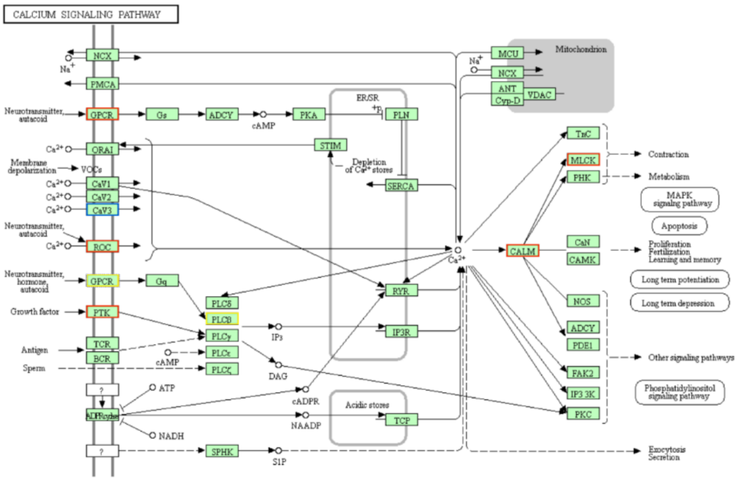

Figure S4. Sketch map of calcium signaling pathway between RBW and MBW group.

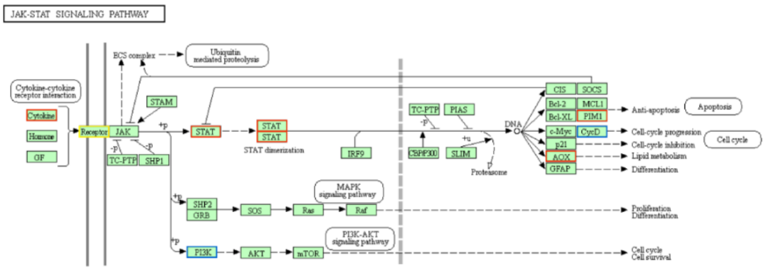

Figure S5. Sketch map of JAK-STAT signaling pathway RBW and MBW group.
